# Is gene therapy for limb ischemia a reality?

**DOI:** 10.1590/1677-5449.190059

**Published:** 2020-05-29

**Authors:** Sang Won Han, Carlos Alberto Vergani, Paulo Eduardo Ocke Reis

**Affiliations:** 1 Universidade Federal de São Paulo – UNIFESP, Departamento de Biofísica, Escola Paulista de Medicina, São Paulo, SP, Brasil.; 2 Universidade Federal de São Paulo – UNIFESP, Centro Interdisciplinar de Terapia Gênica – CINTERGEN, São Paulo, SP, Brasil.; 3 Universidade Federal Fluminense – UFF, Departamento de Cirurgia Geral e Especializada, Rio de Janeiro, RJ, Brasil.

**Keywords:** gene therapy, atherosclerosis, peripheral artery disease, limb ischemia

## Abstract

The concept of angiogenic therapy emerged in the early 1990s. The method employs genes that encode growth factors to promote formation of new vessels and remodeling of collateral vessels. Since the procedure involved in this therapy usually only consists of local injections of vectors, the process is minimally invasive, quick, and simple to perform. However, since the first clinical evidence of the effects of gene therapy with vascular endothelial growth factor (VEGF) was observed in patients with peripheral artery disease, to date only two angiogenic therapy drugs have been approved, one in Russia and another in Japan, which seem a very small number, in view of the large volume of investment made in pre-clinical and clinical studies. After all, can we conclude that angiogenic therapy is a reality?

## INTRODUCTION

Cases of peripheral arterial occlusive disease (PAOD) increase significantly among people more than 70 years old and who have diabetes.^1^^,^^2^ As the global population becomes ever older and engages in bad habits such as inactivity and poor diet acquired in conjunction with modern lifestyles, the prevalence rates of diabetes and morbid obesity have also increased, provoking further increases in the number of patients with PAOD. This disease is silent during its initial phases and it is discovered when the pathology is already advanced, making prevention and treatment difficult. The main symptom of the initial progression of PAOD is pain when walking, termed intermittent claudication. As the disease progresses, there is pain even when at rest, which is more intense at night, and ulcers develop on the lower limbs that do not heal easily. These symptoms are a consequence of progressive reductions in tissue perfusion and are characteristic of critical lower limb ischemia (CLI).^3^

The principal cause of PAOD is atherosclerosis, which can affect arteries all over the body. This is why it is associated with other obstructive arterial diseases (coronary, cerebral, and carotid) and, consequently, the risk of cardiovascular events such as stroke and myocardial infarction increases by around 6% per year in patients with PAOD^4^. According to the Transatlantic Inter-Society Consensus,^2^ approximately 30% of patients with CLI will undergo an amputation, because revascularization and clinical treatments are not feasible in this population, and 25% of these patients will die within 1 year. Each patient’s course is variable and the stage of the disease is symptom-dependent, so prognosis varies from case to case. Patients with PAOD over the age of 50 have worse prognosis within 5 years: approximately 10% will undergo limb amputations and another 10-15% will die from cardiovascular diseases. In summary, all these data indicate worsening prognosis for modern society, which is aging progressively and has unhealthy habits.

Since atherosclerosis is the principal cause of PAOD and of cardiovascular diseases, the clinical treatments for these are similar to those used with patients with cardiovascular ischemia, including medications to reduce lipids, to control hypertension, to prevent platelet aggregation, and of glycemia for diabetic patients with PAOD.^5^ Intermittent claudication is one of the primary initial symptoms of disease that may progress to CLI and the medication most frequently used to treat is cilostazol, which inhibits phosphodiesterase type III and acts as an antiplatelet agent and a vasodilator.^5^ However, pharmacotherapy that acts on the endothelium to provoke vasodilation, angiogenesis, and remodeling of vessels with the aim of improving these patients’ vascular function has demonstrated little benefit, probably because their arteries are already in an environment with atherosclerosis, fibrosis, and calcification, interfering with adequate interaction between the drugs and their receptors.

Currently, the procedures most frequently used to treat CLI are revascularization surgery and percutaneous angioplasty. However, since patients with CLI are, in general, elderly, smokers, and diabetic, approximately 30% of them cannot undergo a vascular procedure despite all of the development that has occurred in the field, and amputation is often the only option. In the United States alone, it is estimated that 120 to 500 lower limb amputations are performed per million inhabitants every year.^2^ Spending on treatments for peripheral arterial diseases in the United States passed 4 billion dollars in 2001,^6^ and the figure is likely to increase annually as risk factors such as diabetes and obesity increase and life expectancy extends.

Faced with the prediction of a continuous increase in the numbers of patients with PAOD and the limitations of conventional treatment options, whether open bypass surgery or angioplasties with balloon angioplasty and stenting to revascularize limbs, gene therapy with genes that express growth factors emerged as a possible solution to these problems at the start of the 1990s. This review article summarizes clinical trials of gene therapy for limb ischemia and offers the authors’ opinions on the future of angiogenic therapy.

## THE CONCEPT OF ANGIOGENIC THERAPY WITH GENES, CELLS, AND PROTEINS

The new proposal is to treat ischemic diseases using growth factors to provoke formation of new vessels and/or remodeling of dysfunctional vessels. These factors, which are proteins, can be administered in their protein form, or via genes or cells that express these factors and the treatment modality is known as therapeutic angiogenesis.^7^ In comparison to surgery, therapeutic angiogenesis is much less invasive and execution is simpler, generally performed by means of a simple injection into the target tissues. Moreover, since the active agents are growth factors, their activities are specific to only those cells that express their receptors and so, as a consequence, gene and protein-based methods should have fewer secondary effects than cell-based therapies, because cells produce a large number of different factors. The most important characteristics of each type of treatment are summarized in Table 1.

**Table 1:** Comparison of angiogenic therapies.

	**Protein therapy** 1 **(PT)**	**Cell therapy** 2 **(CT)**	**Gene therapy** 3 **(GT)**
Complexity of production	+++	+ / ++	+++
Cost of production	++ / +++	+ / ++	++/+++
Stability of drug	+	+	++ / +++
Applicability across different patients	Yes	No	Yes
Durability of therapeutic effects	+	+ / ++	++ / +++
Immunogenicity	- / +	- / +	+ (pDNA, AAV, RV, LV); +++ (AdV)

1 therapy using human proteins;

2 therapy using autologous cells;

3 therapy using human genes; pDNA (plasmid vectors); AAV (adenovirus-associated vector); RV (retroviral vector); LV (lentiviral vector); AdV (adenoviral vector). The signs represent the intensity of the parameters analyzed: high (+++), moderate (++), low (+) or none (-).

The concept of using genes to treat diseases emerged at the end of the 1960s when synthetic biology became a reality with the discovery of the structure of DNA, of genetic codes, and of enzymes for genetic engineering, among others.^8^ However, the first clinical trial with gene therapy was only conducted in 1990, in the United States, after approval of the protocol by the US Federal agency, the Food and Drug Administration (FDA). The trial involved treatment of two patients with severe combined immunodeficiency caused by the deficiency of adenosine deaminase (SCID-ADA).^9^ Since then, 2,597 trials had been approved worldwide, up to 2019, according to the website.^10^

Gene therapy is performed by transferring a vector carrying therapeutic genes to the patient, which can be done directly to the patient (in vivo gene therapy) or using genetically modified cells (ex vivo gene therapy). The majority of clinical gene therapies for PAOD have been in vivo using plasmid (pDNA) or adenovirus vectors (Ad), because they have a good capacity for in vivo transfection (non-viral vectors) or transduction (viral vectors). Adenoviral vectors are more effective for gene transfer than pDNA vectors, but because capsid proteins are very immunogenic, the prior immunoresponse must be controlled for repeated administrations.^11^ Another disadvantage of using Ad is the greater complexity of vector production and quality control, which makes the process more expensive than using pDNA. On the other hand, using pDNA for gene therapy requires greater quantities of the vector or a carrier (for example, liposomes) to compensate for the low efficiency of gene transfer in vivo, but since these vectors are considered much less immunogenic and more stable than viral vectors, there are a considerable number of clinical trials using pDNA.^10^

## CLINICAL TRIALS OF GENE THERAPY FOR LIMB ISCHEMIA

**Figure 1:**
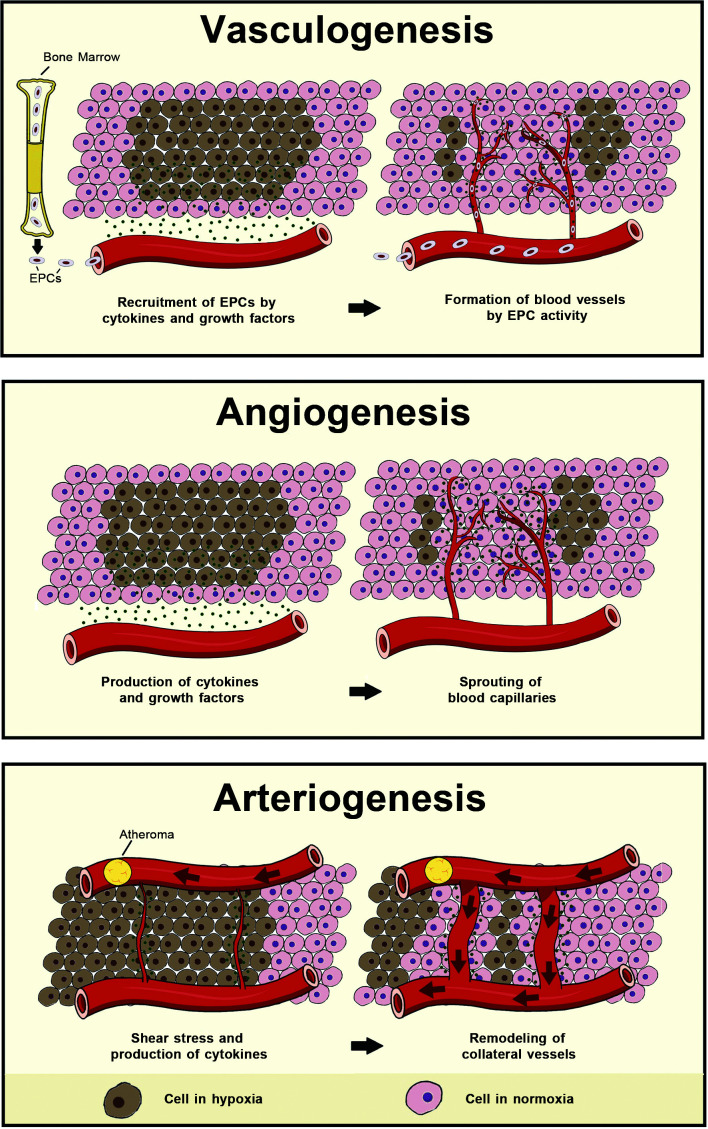
Image illustrating formation and remodeling of vessels in adulthood. Angiogenesis, vasculogenesis, and arteriogenesis are processes that lead to formation and remodeling of vessels in adulthood (the details of these processes are described in the text). EPC: endothelial precursor cell.

**Figure 2:**
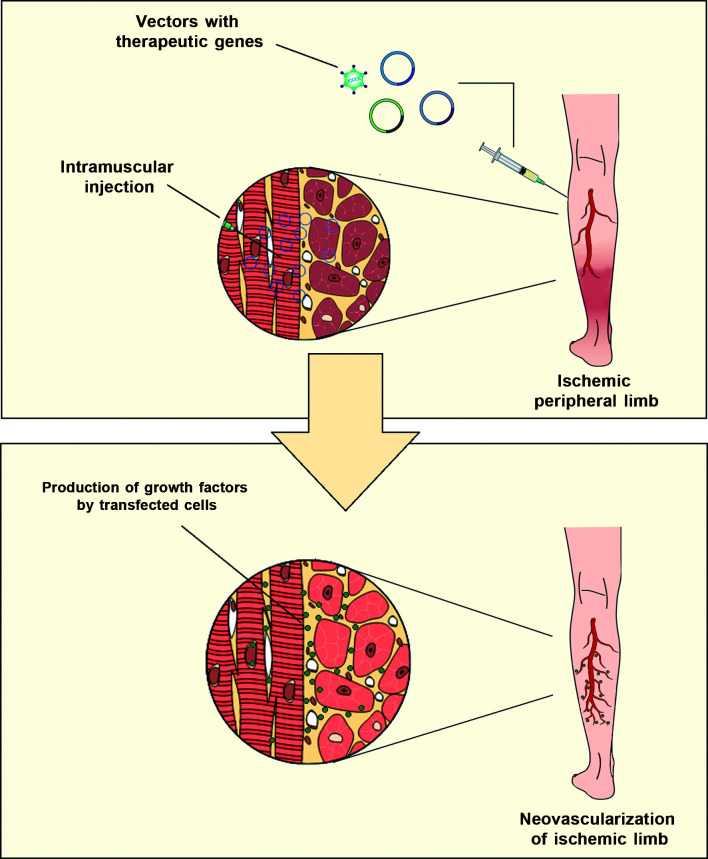
Image illustrating gene therapy for critical limb ischemia with growth factor genes.

Clinical trials of angiogenic therapies conducted to date have used just a single gene at a time to promote formation of new vessels from preexisting vessels by sprouting (angiogenesis) and/or from precursor endothelial cells (vasculogenesis) and/or by remodeling of collateral vessels (arteriogenesis) (Figure 1).^12^ The procedure used for gene therapy is very simple, consisting of intramuscular injections of vectors (Figure 2), but the technology used to create and produce these vectors, that are designed to change the state of functionally altered and pathological tissues, is complex.

The genes most often used for angiogenic therapy in clinical trials include the genes for vascular endothelium growth factor (VEGF),^13^ for fibroblast growth factor (FGF)^14^, for hepatocyte growth factor (HGF)^15^ and for hypoxia inducible factor (HIF-1𝛼)^16^^,^^17^. Details of these factors and the clinical trials involving their genes are described below.

HIF-1𝛼 (hypoxia inducible factor -1𝛼): this factor is constitutively encoded by the human HIF-1𝛼 gene and is located in cytosol. In normoxic conditions, this factor is degraded by ubiquitination, but under hypoxia it translocates to the nucleus and joins with HIF-1ß and other accessory factors to form a protein complex that acts in transcription of more than 60 genes. In general, these genes are linked with angiogenesis (for example, the VEGF gene), erythropoiesis, gluconeogenesis, and vasodilation, whose activities are intimately related to survival under hypoxia.^18^

Use of this gene was tested in a clinical trial named the WALK study in patients with PAOD and intermittent claudication.^16^ In this study, 289 patients received 20 injections of the adenoviral vector Ad2/HIF-1𝛼/VP16 into muscles affected by ischemia. The patients were monitored for 12 months, and changes in walking were observed. The study was double-blind and randomized. Median peak walking time was 0.82 minutes in the placebo group and 0.82 minutes, 0.28 minutes, and 0.78 minutes in experimental groups given doses of 2x10^9^, 2x10^10^, and 2x10^11^ of Ad2/HIF-1𝛼/VP16, respectively. There was no significant difference in ankle-brachial index (ABI) or in quality of life between groups treated with placebo and groups treated with the Ad2/HIF-1𝛼/VP16 vector. Therefore, the authors concluded that the gene therapy investigated in the study was not an effective treatment for patients with intermittent claudication.

FGF-1 (fibroblast growth factor-1): also known as aFGF (acid FGF) is member of the FGF family, which has several biological activities such as endothelial cell proliferation and migration, angiogenesis, cell survival, morphogenesis, and tissue repair, among others. Several activities related to angiogenesis observed in vitro and in vivo studies prompted testing of FGF in patients.^19^^,^^20^ Among the several clinical trials using this growth factor, those conducted using the non-viral 1 FGF-1 vector (NV1FGF) are probably the best known. The NV1FGF vector is a plasmid vector for expression of FGF-1 that was designed and produced by Sanofi-Aventis. Although FGF1 is a non-integrating vector, its expression has persisted in muscle for several weeks in some patients.^21^

The results of the phase I^22^ and II^23^ clinical trials satisfactorily achieved their proposed objectives, enabling progression to phase III.^24^ The phase III study, known as TAMARIS, was a multicenter, double-blind, and randomized study conducted at 171 centers in 30 countries. The 525 patients who participated in the study were in a clinical condition that was inappropriate for revascularization and had ischemic ulcers or gangrene. Hemodynamic parameters for study enrollment were ankle pressure < 70 mmHg and/or toe pressure < 50 mmHg, or transcutaneous oxygen pressure < 30 mmHg. The primary objective of this study was to demonstrate the clinical benefits of NV1FGF in extending survival time to major amputation or death of patients with limb ischemia and non-healing ulcers in whom revascularization was infeasible. The patients received eight intramuscular injections of 0.5 mg of NV1FGF or placebo at approximately 2-week intervals. The numbers of amputations and deaths were not statistically different between groups treated with NV1FGF and placebo, i.e., the therapeutic effect of NV1FGF was not demonstrated.

HGF (hepatocyte growth factor): it is another pleiotropic factor that acts on several physiological activities such as cell proliferation, angiogenesis, morphogenesis, and motility.^25^ Endothelial and smooth muscle cells express the HGF receptor cMet and are the principal cells related to angiogenesis.^26^ Intramuscular injection of pDNA expressing HGF in animals demonstrated angiogenic activity, prompting the first gene therapy clinical trials. Morishita et al. conducted a phase I/IIa clinical trial with 22 patients with peripheral arterial disease or Buerger disease at Fontaine stages IIb to IV with four or eight injections of pDNA expressing HGF (2 mg at four sites or 4 mg at eight sites) on days 1 and 28. The main findings that led to continuation of the study were increased ABI and reductions in ulcers and pain.^27^ A phase III, randomized, double-blind, and placebo-controlled study was then conducted with 44 patients.^28^ The primary objectives were improvement of pain at rest or reductions in ulcers in patients with ulcers, and the secondary objectives were improvement of ABI, amputation rates, and quality of life. There was a significant improvement in primary objectives and quality of life without significant negative effects, but the amputation rate and ABI did not improve. This gene therapeutic drug (Collategene) was conditionally approved in Japan in 2019.

The BM202 vector is a plasmid that expresses two isoforms of HGF.^29^ Oddly, expression of both isoforms led to a significant improvement in ABI, which was not observed in the study by Shigematsu et al.^28^ Based on that study, a phase II study was approved and is ongoing.

VEGF (vascular endothelial growth factor): this is the growth factor that has been most studied for angiogenic therapy. There are four main isoforms (VEGF A, B, C, and D), and alternative splicing, which is a process for alternative substitution of introns and joining of exons to form a new mRNA, leads to formation of additional isoforms. Human VEGF A can form VEGF121, VEGF165, VEGF189, and VEGF206, the first two of which are most used for gene therapy. It was recently observed that the native or modified forms of the VEGF D isoform, which act in lymphangiogenesis, can also actively promote angiogenesis.^30^^,^^31^ The receptors of VEGF are Flt-1 and Flk-1, also known as VEGFR-1 and VEGFR-2, respectively. The VEGFR-1 receptor binds to VEGF A and B, while VEGFR-2 only binds to VEGF A.^32^ The VEGFR receptors act in conjunction with neutropilin-1, which is considered a coreceptor of VEGF. Both VEGFR-1 and VEGFR-2 are present in endothelial cells.

The pioneering work with angiogenic therapy was initiated in 1994 by Jeffrey Isner and his team, showing that formation of vessels could be stimulated with a plasmid vector expressing VEGF165 (phVEGF165) at the tip of a hydrogel catheter.^33^ Years later, the same group conducted another clinical study, administering phVEGF165 directly into the ischemic limbs of patients with PAOD. In that study, two applications of 2 mg of phVEGF165 were administered with a 2-week interval.^34^ Mean ABI increased significantly and formation of collateral vessels was demonstrated by angiography with contrast and magnetic resonance angiography. There was significant improvement in healing of ulcers in several patients. In addition to demonstrating the efficacy of angiogenic therapy for treatment of ischemic diseases, this study also demonstrated use of the plasmid vector for ischemic diseases, which is a vector that is simpler to design and produce than other vectors, and, in combination with the simple method of administration, this marked the start of a new era in angiogenic therapy.

A number of different clinical trials of angiogenic therapy with VEGF have been conducted.^10^ Although the initial results were encouraging, studies conducted later with larger sample sizes reported controversial results. For example, a phase II study by Kusumanto et al.,^35^ in which phVEGF165 was administered to 54 diabetic patients with critical limb ischemia, was unable to demonstrate improvements in terms of reductions in amputation at 100 days, despite having demonstrated significant improvements in pain, ulcer healing, and ABI in some patients. Another phase II study, this one using the adenoviral vector VEGF121 (AdVEGF121), was conducted with 105 patients with peripheral arterial disease.^36^ The primary objective of increasing walking time at 12 weeks was not achieved, and edema was observed in several patients after administration of the vector. The study’s conclusion was that intramuscular administration of the AdVEGF121 vector was not associated with improved exercise performance or quality of life. These and other clinical trials of angiogenic therapy with VEGF reported controversial efficacy results.

﻿Despite these controversies, in 2011, a plasmid VEGF165 vector, given the commercial name Neovasculgen, was approved in Russia for treatment of patients with limb ischemia after a phase IIb/III clinical trial with 100 patients. Patients in this randomized study were treated twice with 1.2 mg of the pCMV-vegf165 vector with a 14-day interval, or were given conventional treatment in the control group.^37^ The distance walked without pain increased 110.4%, 167.2%, and 190.8%, 6 months, 1 year, and 2 years after treatment, respectively. Additionally, ABI and blood flow velocity also improved significantly. The authors concluded the article stating that treatment with pCMV-vegf165 is an effective method for treatment of moderate to severe claudication caused by CLI.

Why are the promising results observed in pre-clinical trials not replicated at the same proportion in clinical trials?

In general, pre-clinical studies of gene therapy for limb ischemia have employed mice for testing. Ischemia is induced surgically by closure of the distal and proximal femoral artery followed by removal of the arterial segment. In some models, the collateral arteries are also closed to induce severe ischemia.^38^ As a result, local circulation is drastically reduced, to the extent that no flow can be detected with laser Doppler perfusion imaging (LDPI) soon after surgery. Although ischemia is rapidly established by this procedure, its pathophysiology is very different from that caused by atherosclerosis, which is a slow process that occurs with the deposition of fats and cells on the artery wall. It is, therefore, a sub-representative model of the human disease.

Furthermore, the mouse strain most often used in these studies is C57/Bl6, possibly because of the availability of a large amount of information about this strain and the low cost of obtaining and maintaining these mice. However, when these studies are compared with other similar studies conducted with the Balb/c strain, there is a notable difference in the degree of ischemia and the results of the treatments applied. Nowadays, it is known that this occurs because there is a significant difference in the vascular anatomy of these mouse strains due to the genetic variation^39^ and because the Balb/c lineage is much more sensitive to ischemia than the C57/Bl6 strain. If the degree of ischemia generated and the responses to angiogenic therapies are so different between two mouse strains, how great a difference might there be between mice and human patients?

Emergence of PAOD is a consequence of the interplay between risk factors and the genetic factors that each individual carries. Therefore, pathophysiologic variations between patients are great, whereas in the animal model the variation is minimal because the environment has been conditioned to reduce variations as much as possible, in order to facilitate interpretation of the results. What are the chances that a drug tested successfully in an animal model under these conditions will have the same benefits for patients with PAOD?

## PROSPECTS FOR ANGIOGENIC TREATMENTS FOR LIMB ISCHEMIA

To date, just two angiogenic therapy drugs have been approved; Neovasculgen in Russia in 2011, and Collategene in Japan in 2019. It is important to point out that both drugs are based on plasmid vectors carrying growth factors that act on endothelial cells to promote angiogenesis. As mentioned above, vectors derived from plasmids are simply DNA molecules, so there is no danger that they will replicate in the body,^40^ which is a great advantage over viral vectors. Furthermore, production and quality control are simpler and less expensive than with viral vectors.

However, the effectiveness of these drugs is still questionable, because there are conflicting clinical trial results. Formation of vessels is a complex process involving proliferation and differentiation of precursor cells under the control of many regulatory molecules.^41^ Therefore, using a single factor that specifically acts on endothelial cells is possibly insufficient to form a mature vessel in an ischemic and inflamed environment.

The ideal angiogenic treatment for limb ischemia would be one that can act on all or a majority of the cells and molecules that participate in angiogenesis and control of inflammation. Below, we suggest some ways to achieve this ideal treatment.

1. Using more angiogenic genes: to date, all of the clinical trials of gene therapy have been conducted using a single gene per trial. Since many genes are essential to angiogenesis, use of more than one of these genes could produce better therapeutic effects. In practice, this is possible using bicistronic or tricistronic vectors or cotransfection of several monocistronic vectors to express several genes simultaneously in the target tissues;
2. Using genetically modified stem cells: stem cells have the plasticity to differentiate into other cell types and express several pro-angiogenic growth factors. Of the known types of stem cells, mesenchymal stem cells are the most useful for clinical use, because of the ease of obtaining them in large quantities from bone marrow and fat and their capacity to promote angiogenesis.^42^ However, guiding their differentiation into a cell type and a type of activity is primarily dependent on the cells’ microenvironment. Genetic modifications with the vectors used for gene therapy could be used to direct pro-angiogenic differentiation and activity, performing ex vivo gene therapy;^43^
3. Using hematopoiesis genes: hematopoiesis, ischemia, and inflammation are intimately interconnected biological processes. Ischemia and inflammation stimulate hematopoiesis to produce more blood cells, and monocytes and macrophages are the principal elements that participate in control of inflammation and angiogenesis.^44^ Monocyte and macrophage subpopulations can be classified according to their inflammatory activities as proinflammatory or anti-inflammatory.^45^ These subpopulations can promote or inhibit angiogenesis and fibrogenesis for repair of ischemic and inflamed tissues. The genes that code for colony stimulating factors such as granulocyte-macrophage colony-stimulating factor (GM-CSF), granulocyte colony-stimulating factor (G-CSF), and macrophage colony-stimulating factor (M-CSF), and interleukins such as IL4 and IL13 participate in directing and increasing these subpopulations of monocytes and macrophages. Therefore, used correctly, these genes could lead to formation and remodeling of vessels in a more efficient manner in the ischemic and inflamed environment.^46^^,^^47^

In summary, it can be stated that gene therapy for limb ischemia is already a clinical reality, since the two gene therapy drugs are already available on the market. The efficacy of the drugs covered in this review is still questionable, but what is important is that the history of gene therapy shows that scientific and technological research are overcoming the barriers of the unknown and are enabling creation of new, more effective, and safer drugs with natural or synthetic genetic materials in combination with nanocarriers or genetically modified viruses. In Brazil, the regulatory framework for advanced therapies including gene therapy, cell therapy, and tissue engineering was approved this year by the National Agency for Sanitary Vigilance (Agência Nacional de Vigilância Sanitária - ANVISA).^48^ As a result, gene therapy drugs that have already been approved or are under clinical trials in other countries can be sold or tested in Brazil. In view of this, it is important that Brazilian medicine is prepared to take advantage of these technologies and their products, by keeping up to date.
