# Supplementary material for: Is gene therapy for limb ischemia a reality?
Source: J Vasc Bras. 2020 May 29;19:e20190059. doi: 10.1590/1677-5449.190059 (PMC8202161; doi:10.1590/1677-5449.190059)
Supplement: Translation

# Translation: Terapia gênica de isquemia de membro é uma realidade?

**Authors:** Sang Won Han, Carlos Alberto Vergani, Paulo Eduardo Ocke Reis

**Affiliations:** 1 Universidade Federal de São Paulo – UNIFESP, Departamento de Biofísica, Escola Paulista de Medicina, São Paulo, SP, Brasil.; 2 Universidade Federal de São Paulo – UNIFESP, Centro Interdisciplinar de Terapia Gênica – CINTERGEN, São Paulo, SP, Brasil.; 3 Universidade Federal Fluminense – UFF, Departamento de Cirurgia Geral e Especializada, Rio de Janeiro, RJ, Brasil.

**Keywords:** terapia gênica, aterosclerose, doença arterial periférica, isquemia de membro

## Abstract

O conceito de terapia angiogênica surgiu no início da década de 90, o que pode ser feito com genes que codificam fatores de crescimento para promover a formação de novos vasos e o remodelamento de vasos colaterais. Como o procedimento dessa terapia geralmente consiste em apenas injeções locais de vetores, esse processo é pouco invasivo, rápido e de simples realização. Entretanto, desde as primeiras evidências clínicas do efeito de terapia gênica com o fator de crescimento de endotélio vascular (*vascular endothelial growth factor*, VEGF) vistos nos pacientes com doença arterial obstrutiva periférica até hoje, apenas dois fármacos de terapia angiogênica foram aprovados, um na Rússia e outro no Japão, o que parece um número muito pequeno diante do grande número de investimentos feitos por meio de estudos pré-clínicos e clínicos. Afinal, podemos considerar que a terapia angiogênica já é uma realidade?

## INTRODUÇÃO

Os casos de doença arterial obstrutiva periférica (DAOP) aumentam significativamente em indivíduos com mais de 70 anos de idade e com diabetes^1^^,^^2^. Com a população mundial cada vez mais velha e apresentando maus hábitos adquiridos com a vida moderna, como sedentarismo e alimentação inadequada, a prevalência de diabetes e de obesidade mórbida também aumentou, promovendo ainda mais o aumento de pacientes com DAOP. É uma doença silenciosa na fase inicial; portanto, sua descoberta ocorre quando o quadro patológico está bem avançado, o que dificulta a prevenção e o tratamento também. O principal sintoma no início da evolução da DAOP é a dor durante a deambulação, o que caracteriza a claudicação intermitente. Conforme progride, a doença evolui para dor mesmo em repouso, mais proeminente à noite, e úlceras nos membros inferiores que não cicatrizam facilmente; esses sintomas são consequências de diminuição progressiva da perfusão tecidual e são características de isquemia crítica de membros inferiores (ICMI)^3^.

A principal causa da DAOP é a aterosclerose, que pode afetar artérias de todo o corpo. Por isso, está associada a outras doenças arteriais obstrutivas (coronariana, cerebral e carotídea) e, consequentemente, o risco de eventos cardiovasculares como o derrame cerebral e o infarto do miocárdio aumenta em cerca de 6% por ano em pacientes com DAOP^4^. De acordo com o *Transatlantic Inter-Society Consensus* 2, aproximadamente 30% dos pacientes com ICMI sofrerão amputação, porque os tratamentos de revascularização e clínico não são factíveis para essa população, e 25% desta morrerá dentro de um ano. A evolução de cada paciente é variável e o estágio da doença é sintoma-dependente; portanto, o prognóstico é variável de acordo com cada caso. Pacientes portadores de DAOP com mais de 50 anos têm um prognóstico pior dentro de 5 anos: aproximadamente 10% sofrerão amputação de membros e outros 10-15% deverão morrer em decorrência de doenças cardiovasculares. Em resumo, esse conjunto de dados indica um prognóstico pior para a sociedade moderna, que envelhece cada vez mais com hábitos não saudáveis.

Como aterosclerose é a principal causa da DAOP e das doenças cardiovasculares, os tratamentos clínicos dessas doenças são similares aos usados nos pacientes com isquemia cardiovascular, como o uso de medicamentos para a redução de lipídios, o controle de hipertensão, o uso de agentes que evitam a agregação plaquetária e o controle de glicemia para os pacientes diabéticos com DAOP^5^. Para claudicação intermitente (um dos principais sintomas iniciais da doença que pode evoluir para ICMI), o medicamento mais utilizado é o cilostazol, que é o inibidor de fosfodiesterase tipo III e atua como antiplaquetário e vasodilatador^5^. Entretanto, a farmacoterapia que atua no endotélio para vasodilatação, angiogênese e remodelamento de vasos a fim de melhorar a função vascular desses pacientes tem trazido pouco benefício porque, provavelmente, as artérias desses pacientes já estão no ambiente de aterosclerose, fibrose e calcificação que dificulta a interação adequada entre fármacos e seus receptores.

Atualmente, os procedimentos mais usados para tratar ICMI são a cirurgia de revascularização e a angioplastia percutânea. Entretanto, como os pacientes com ICMI são, em geral, idosos, fumantes e diabéticos, aproximadamente 30% desses pacientes não podem ser submetidos ao procedimento vascular apesar de todos os avanços nessa área, sendo a amputação muitas vezes a única opção. Somente nos Estados Unidos, estima-se que ocorram entre 120 e 500 amputações de membros inferiores por milhão de habitantes por ano^2^. Gastos para tratamentos de doenças arteriais periféricas nos Estados Unidos chegaram a mais que 4 bilhões de dólares em 2001^6^, e esse valor deve aumentar a cada ano devido ao aumento de fatores de risco como diabetes, obesidade e aumento da expectativa de vida.

Diante da projeção de um aumento contínuo de pacientes com DAOP e das limitações de opções terapêuticas convencionais, seja cirurgia aberta por *bypass* ou angioplastias com balão de angioplastia e stent para revascularização do membro, a terapia gênica com genes que expressam os fatores de crescimento surgiu como uma solução desses problemas no início da década de 90. Esta revisão sumariza os ensaios clínicos de terapia gênica para isquemia de membro e coloca opiniões sobre o futuro de terapia angiogênica sob o olhar dos autores.

## CONCEITO DE TERAPIA ANGIOGÊNICA COM GENES, CÉLULAS E PROTEÍNAS

A nova aposta de tratar doenças isquêmicas é a utilização de fatores de crescimento para promover a formação de novos vasos e/ou o remodelamento de vasos pouco funcionais. Esses fatores, que são de natureza proteica, podem ser administrados na forma proteica, gênica ou via células que expressam esses fatores; essa modalidade terapêutica é conhecida por angiogênese terapêutica^7^. Em relação a cirurgias, a angiogênese terapêutica é muito menos invasiva e de execução mais simples, sendo geralmente é feita por meio de uma simples injeção no tecido alvo. Além disso, como os princípios ativos são fatores de crescimento, suas ações são específicas somente nas células que expressam seus receptores; consequentemente, as terapias gênica e proteica deveriam provocar menos efeitos secundários que a terapia celular, porque esta última produz um grande número de fatores. As principais características de cada terapia estão mostradas na Tabela 1.

**Tabela 1:** Comparação entre as terapias angiogênicas.

	**Terapia proteica** 1 **(TP)**	**Terapia celular** 2 **(TC)**	**Terapia gênica** 3 **(TG)**
Complexidade da produção	+++	+ / ++	+++
Custo de produção	++ / +++	+ / ++	++/+++
Estabilidade do fármaco	+	+	++ / +++
Aplicabilidade entre diferentes pacientes	Sim	Não	Sim
Durabilidade do efeito terapêutico	+	+ / ++	++ / +++
Imunogenicidade	- / +	- / +	+ (pDNA, AAV, RV, LV); +++ (AdV)

1 terapia com proteínas humanas;

2 terapia com células autólogas;

3 terapia com genes humanos; pDNA (vetor plasmidial); AAV (vetor do vírus adeno-associado); RV (vetor retroviral); LV (vetor lentiviral); AdV (vetor adenoviral). Os sinais representam a intensidade processual entre os parâmetros analisados: muito (+++), médio (++), pouco (+) ou nada (-).

O conceito de tratar doenças com genes surgiu no final da década de 1960, quando a biologia sintética se tornou realidade com a descoberta da estrutura de DNA, dos códigos genéticos e das enzimas para engenharia genética, entre outros^8^. Entretanto, o primeiro ensaio clínico de terapia gênica ocorreu somente em 1990, nos EUA, após aprovação do protocolo pelo órgão federal *Food and Drug Administration* (FDA), o qual foi realizado para tratamento de duas pacientes com *severe combined immunodeficiency* causada pela deficiência de *adenosine deaminase* (SCID-ADA)^9^. Desde esse ensaio clínico, 2.597 ensaios já foram aprovados até 2019 no mundo, segundo o site^10^. 

A terapia gênica é realizada por meio da transferência de um vetor com gene terapêutico ao paciente, que pode ser feita diretamente ao paciente (terapia gênica *in vivo*) ou com as células modificadas geneticamente (terapia gênica *ex vivo*). A maioria das terapias gênicas clínicas para DAOP têm sido feitas *in vivo* usando os vetores de plasmídeo (pDNA) ou de adenovírus (Ad), porque estes têm uma boa capacidade de transfecção (vetor não viral) ou transdução (vetor viral) *in vivo*. Os vetores Ad são mais eficazes que os pDNA para transferência gênica, mas, como as proteínas capsídicas são muito imunogênicas, a resposta imune anterior precisa ser controlada para aplicações repetidas^11^. Outra desvantagem do uso de Ad é a maior complexidade da produção dos vetores e do controle de qualidade, que gera alto custo em relação ao pDNA. Por outro lado, o uso de pDNA para terapia gênica requer maior quantidade deste ou um carreador (por exemplo, lipossoma) para compensar a baixa eficiência da transferência gênica *in vivo*, mas, como esse vetor é considerado bem menos imunogênico e mais estável que vetores virais, há um bom número de estudos clínicos usando pDNA^10^.

## ESTUDOS CLÍNICOS DE TERAPIA GÊNICA PARA ISQUEMIA DE MEMBRO

Estudos clínicos de terapia angiogênica realizados até hoje têm usado apenas um único gene por vez para promover a formação de novos vasos a partir dos vasos pré-existentes por brotamento (angiogênese) e/ou a partir de células precursoras endoteliais (vasculogênese) e/ou remodelamento de vasos colaterais (arteriogênese) (Figura 1)^12^. O procedimento de terapia gênica é bastante simples, consistindo em injeção intramuscular de vetores (Figura 2), mas a tecnologia para criação e produção desses vetores, que visam alterar um estado tecidual funcionalmente desarranjado e patológico, é complexa.

**Figura 1:** Ilustração de formação e remodelamento de vasos na fase adulta. Angiogênese, vasculogênese e arteriogênese são processos que levam à formação e ao remodelamento de vasos na fase adulta (os detalhes desses processos estão no texto). EPC: célula progenitora endotelial.

**Figura 2:** Ilustração de terapia gênica com genes de fator de crescimento para isquemia crítica de membro.

Entre os genes mais usados para terapia angiogênica nos estudos clínicos estão os genes do fator de crescimento de endotélio vascular (VEGF)^13^, do fator de crescimento de fibroblasto (FGF)^14^, do fator de crescimento de hepatócitos (HGF)^15^ e do fator induzível por hipóxia (HIF-1𝛼)^16^^,^^17^. Os detalhes desses fatores e os estudos clínicos com esses genes são descritos abaixo.

HIF-1𝛼 (*hypoxia inducible factor -1𝛼*, fator induzível por hipóxia 1𝛼): esse fator é codificado pelo gene HIF-1𝛼 humano constitutivamente e se localiza no citosol. Em condições de normóxia, esse fator é degradado via ubiquitinação, mas sob hipóxia se transloca para o núcleo e se associa ao HIF-1ß e a outros fatores acessórios para formar um complexo proteico que atua na transcrição de mais de 60 genes. Em geral, esses genes estão ligados à angiogênese (por exemplo, o gene do VEGF), eritropoese, neoglicogênese e vasodilatação, cujas atividades são intimamente ligadas à sobrevivência sob hipóxia^18^.

O uso desse gene foi testado num estudo clínico denominado WALK em pacientes com DAOP e claudicação intermitente^16^. Para esse estudo, 289 pacientes receberam 20 injeções do vetor adenoviral Ad2/HIF-1𝛼/VP16 nos músculos afetados por isquemia. Esses pacientes foram monitorados durante 12 meses, observando-se a mudança de caminhada. Foi um estudo de duplo-cego e randomizado. O pico mediano do tempo de caminhada foi de 0,82 minutos no grupo placebo e de 0,82 minutos, 0,28 minutos e 0,78 minutos para os grupos que receberam 2x10^9^, 2x10^10^ e 2x10^11^ de doses do Ad2/HIF-1𝛼/VP16, respectivamente. Não houve diferença significativa no índice de tornozelo-braquial (ITB) e na qualidade de vida entre os grupos tratados com placebo e os grupos tratados com o vetor Ad2/HIF-1𝛼/VP16. Assim, os autores concluíram que a terapia gênica desse estudo não foi efetiva no tratamento de pacientes com claudicação intermitente.

FGF-1 (*fibroblast growth factor-1*, fator de crescimento de fibroblasto-1): também conhecido por aFGF (FGF ácido), faz parte da família de FGF, a qual atua em várias atividades biológicas como proliferação e migração da célula endotelial, angiogênese, sobrevivência celular, morfogênese e reparo tecidual, entre outras. Várias atividades ligadas à angiogênese observadas *in vitro* e *in vivo* levaram ao uso do FGF nos pacientes para testes^19^^,^^20^. Entre os ensaios clínicos com esse fator de crescimento, os conduzidos com o vetor *non-viral* 1 FGF-1 (NV1FGF) são provavelmente os mais conhecidos. O NV1FGF é um vetor de plasmídeo para expressão de FGF-1 que foi desenhado e produzido por Sanofi-Aventis. Apesar de ser um vetor não integrativo, a expressão de FGF1 tem persistido no músculo por várias semanas em alguns pacientes^21^.

Os resultados dos estudos clínicos de fase I^22^ e II^23^ atingiram os objetivos propostos satisfatoriamente, o que permitiu avançar para a fase III^24^. O estudo de fase III, conhecido por TAMARIS, foi um estudo multicêntrico realizado em 171 centros de 30 países, duplo-cego e randomizado. Os 525 pacientes que participaram desse estudo estavam com a condição clínica inadequada para revascularização e com úlcera isquêmica ou gangrena. Os parâmetros hemodinâmicos para inclusão no estudo foram pressão do tornozelo < 70 mmHg e/ou do dedo < 50 mmHg, ou pressão de oxigênio transcutânea < 30 mmHg. O principal objetivo desse estudo foi mostrar o benefício clínico do NV1FGF em retardar o tempo de amputação maior ou a morte dos pacientes com isquemia de membro e úlcera não cicatrizante cuja revascularização não era possível. Esses pacientes receberam oito injeções intramusculares de 0,5 mg de NV1FGF ou placebo com um intervalo aproximado de 2 semanas. Os números de amputação ou morte entre os grupos tratados com NV1FGF e placebo não foram diferentes estatisticamente, ou seja, o efeito terapêutico do NV1FGF não foi evidenciado.

HGF (*hepatocyte growth factor,* fator de crescimento de hepatócitos): é outro fator pleiotrópico que atua em diversas atividades fisiológicas como proliferação celular, angiogênese, morfogênese e motilidade^25^. As células endoteliais e musculares lisas expressam o receptor de HGF, cMet, e são as principais células ligadas à angiogênese^26^. Injeção intramuscular do pDNA expressando HGF nos animais tem evidenciado a atividade angiogênica, o que motivou os primeiros estudos clínicos de terapia gênica. Morishita et al. realizaram o estudo clínico de fase I/IIa com 22 pacientes com doença arterial periférica ou doença de Buerger nos estágios Fontaine IIb a IV com quatro ou oito injeções de pDNA expressando HGF (2 mg em quatro locais ou 4 mg em oito locais) nos dias 1 e 28. Aumento de ITB, diminuição de úlcera e dor foram os principais resultados que motivaram a continuidade desse estudo^27^. O estudo de fase III, randomizado, duplo-cego e placebo-controlado foi realizado com 44 pacientes^28^. Os objetivos primários foram o melhoramento de dor em repouso ou a redução de úlcera nos pacientes com úlceras, e os objetivos secundários foram a melhora do ITB, da taxa de amputação e da qualidade de vida. Houve melhora significativa dos objetivos primários e da qualidade de vida sem efeitos negativos significativos, mas a taxa de amputação e o ITB não sofreram melhora. Este medicamento de terapia gênica, Collategene, recebeu aprovação condicional no Japão em 2019.

O BM202 é um vetor de plasmídeo que expressa duas isoformas de HGF^29^. Curioso é que a expressão das duas isoformas levou a uma melhora significativa do ITB, o que não foi visto no estudo do Shigematsu et al.^28^. Baseado nesse estudo, um estudo de fase II foi aprovado e está em andamento.

VEGF (*vascular endothelial growth factor,* fator de crescimento endotelial vascular): é o fator de crescimento mais estudado para fins de terapia angiogênica. Há quatro principais isoformas (VEGF A, B, C e D), e o *splicing* alternativo, que é um processo de remoção alternativa de íntrons e de junção de éxons para formar um novo mRNA, leva à formação de mais isoformas. O VEGF A humano pode formar VEGF121, VEGF165, VEGF189 e VEGF206, sendo que as duas primeiras isoformas são as mais usadas para terapia gênica. Recentemente foi visto que as formas nativas ou modificadas da isoforma VEGF D, que atua em linfangiogênese, também podem promover angiogênese ativamente^30^^,^^31^. Os receptores de VEGF são Flt-1, Flk-1, que também são denominados de VEGFR-1 e VEGFR-2, respectivamente. O VEGFR-1 se liga a VEGF A e B, enquanto que o VEGFR-2 se liga a apenas VEGF A^32^. Os VEGFRs atuam junto com a neutropilina-1, que é considerada a correceptora do VEGF. Nas células endoteliais, os VEGFR-1 e VEGFR-2 estão presentes.

O trabalho pioneiro de terapia angiogênica foi iniciado em 1994 por Jeffrey Isner e seu grupo, em que a formação de vasos pode ser estimulada com a aplicação de um vetor de plasmídeo com VEGF165 (phVEGF165) na ponta de um cateter de hidrogel^33^. Anos depois, o mesmo grupo realizou outro estudo clínico aplicando phVEGF165 diretamente nos membros isquêmicos dos pacientes com DAOP. Nesse estudo, foram realizadas duas aplicações de 2 mg de phVEGF165 com um intervalo de 2 semanas^34^. O índice médio do ITB aumentou significativamente e a formação de vasos colaterais foi evidenciada por angiografia com contraste e angiografia de ressonância magnética. Houve melhora significativa da cicatrização de úlceras em vários pacientes. Esse trabalho, além de ter demonstrado a eficácia de terapia angiogênica no tratamento de doenças isquêmicas, revelou que a utilização do vetor de plasmídeo, que é um vetor de desenho e produção simples em relação a outros vetores, e a forma simples de aplicação marcaram a nova era da terapia angiogênica.

Vários estudos clínicos de terapia angiogênica com VEGF têm sido realizados^10^. Apesar dos resultados empolgantes verificados inicialmente, outros estudos que vieram posteriormente, com tamanhos de amostras maiores, mostraram resultados controversos. Por exemplo, um estudo de fase II realizado pelo Kusumanto et al.^35^ aplicando phVEGF165 em 54 pacientes diabéticos com isquemia crítica de membro não conseguiu demonstrar melhora na redução de amputação em 100 dias, apesar de ter demonstrado melhora significativa de dor, cicatrização de úlcera e do ITB em alguns pacientes. Outro estudo de fase II, mas usando o vetor adenoviral VEGF121 (AdVEGF121), foi realizado em 105 pacientes com doença arterial periférica^36^. O objetivo primário de aumentar o tempo de caminhada em 12 semanas não foi alcançado, e foi observado edema em vários pacientes após a aplicação do vetor. A conclusão do estudo é que uma aplicação intramuscular do vetor AdVEGF121 não estava associada com a melhora de performance de exercício e da qualidade de vida. Esses e outros estudos clínicos de terapia angiogênica com VEGF mostraram resultados controversos de efetividade.

﻿Apesar dessas controvérsias, em 2011 o vetor plasmidial com VEGF165, cujo nome comercial é Neovasculgen, foi aprovado na Rússia para tratamento de pacientes com isquemia de membro após um estudo clínico de fase IIb/III com 100 pacientes. Nesse estudo randomizado, os pacientes foram tratados com 1,2 mg do vetor pCMV-vegf165, duas vezes com intervalo de 14 dias, ou com tratamento convencional para o grupo controle^37^. A distância de caminhada sem dor aumentou 110,4%, 167,2% e 190,8% após 6 meses, 1 ano e 2 anos de tratamento, respectivamente. Além disso, o ITB e a velocidade do fluxo sanguíneo também tiveram melhoria significativa. Os autores concluem o artigo dizendo que o tratamento com o pCMV-vegf165 é um método efetivo para o tratamento de claudicação moderada a severa causada por ICMI.

Por que os resultados promissores vistos nos ensaios pré-clínicos não se repetem na mesma proporção nos ensaios clínicos?

Os estudos pré-clínicos de terapia gênica para isquemia de membro, em geral, têm se realizado usando camundongos para testes. A isquemia é induzida cirurgicamente com fechamento da porção distal e proximal da artéria femoral seguido de remoção dessa extensão arterial. Em alguns modelos, as artérias colaterais também são fechadas para induzir isquemia severa^38^. Com isso, a circulação local se reduz drasticamente a ponto de não se verificar nenhuma pelo *laser* Doppler *perfusion imaging* (LDPI) logo após cirurgia. Embora a isquemia seja restabelecida rapidamente por esse procedimento, a fisiopatologia dessa isquemia é muito diferente daquela gerada por aterosclerose, a qual é um processo lento que ocorre com acúmulo de gorduras e células na parede da artéria. Portanto, é um modelo muito pouco representativo da doença humana.

Além disso, a linhagem de camundongos mais usada nesses estudos é a C57/Bl6, talvez devido à disponibilidade de uma grande quantidade de informações sobre essa linhagem e ao baixo custo para obtenção e manutenção. No entanto, quando esses estudos são comparados com outros estudos similares realizados com a linhagem Balb/c, observa-se diferença notável no grau de isquemia e nos resultados das terapias empregadas. Hoje se sabe que isso ocorre porque a anatomia vascular entre essas linhagens de camundongo tem uma diferença significativa em decorrência da variação genética^39^ e porque a linhagem Balb/c é bem mais sensível à isquemia do que a C57/Bl6. Se o grau de isquemia gerada e das respostas da terapia angiogênica entre as linhagens de camundongo tem uma grande diferença, quanto seria essa diferença entre camundongos e pacientes humanos?

O surgimento da DAOP é consequência do jogo entre os fatores de risco, além do fator genético que cada um carrega. Portanto, a variação fisiopatológica de cada paciente é muito variável, enquanto que no modelo animal essa variação é mínima porque o ambiente foi condicionado para reduzir ao máximo as variações a fim de facilitar a interpretação de resultados. Qual é a chance de uma droga testada com sucesso num modelo animal nessas condições trazer o mesmo benefício aos pacientes com DAOP?

## PERSPECTIVAS DE TERAPIAS ANGIOGÊNICAS PARA ISQUEMIA DE MEMBRO

Até o momento, apenas dois fármacos de terapia angiogênica foram aprovados: Neovasculgen, foi aprovado na Rússia em 2011, e Collategene no Japão. É importante notar que ambos fármacos são construídos com vetores de plasmídeo carreando os fatores de crescimento que atuam nas células endoteliais para promoção de angiogênese. Como foi comentado anteriormente, os vetores derivados de plasmídeo são apenas moléculas de DNA; portanto, não há perigo de se replicarem no organismo^40^, o que é uma grande vantagem sobre os vetores virais. Além disso, o custo para produção e controle de qualidade é mais simples e menos custoso que o dos vetores virais.

Entretanto, a efetividade desses fármacos ainda é questionável, porque há controvérsias nos resultados de ensaios clínicos. A formação de vasos é um processo complexo que envolve multiplicação e diferenciação de células precursoras sob o controle de várias moléculas regulatórias^41^. Assim, o uso de um único fator que atua especificamente em células endoteliais talvez não seja suficiente para formar um vaso maduro num ambiente isquêmico e inflamado.

A terapia angiogênica ideal para isquemia de membro seria aquela que consegue atuar em todas ou na maioria das células e moléculas que participam da angiogênese e do controle da inflamação. Abaixo sugerimos algumas formas de alcançar essa terapia ideal.

1. Uso de mais genes angiogênicos: até hoje todos os ensaios clínicos de terapia gênica têm sido realizados utilizando apenas um único gene por vez. Como há vários genes essenciais em angiogênese, o uso de mais de um desses genes poderá trazer melhores efeitos terapêuticos. Na prática, isso é factível usando vetores bi- ou tricistrônicos ou cotransfecção de vários vetores monocistrônicos para expressar vários genes simultaneamente no tecido alvo;
2. Uso de células-tronco modificadas geneticamente: as células-tronco têm plasticidade para se diferenciar em outros tipos celulares e expressam vários fatores de crescimento pró-angiogênicos. Entre os tipos de células-tronco conhecidos, as células-tronco mesenquimais são as mais adequadas para uso clínico, devido à facilidade de obtenção em grande quantidade da medula óssea e da gordura, e capacidade de promover angiogênese^42^. Entretanto, o direcionamento da diferenciação a um tipo celular e a um tipo de atividade depende principalmente do microambiente onde a célula se encontra. Modificações genéticas com os vetores usados para terapia gênica podem ser usadas para direcionar a diferenciação e a atividade pró-angiogênica e realizar terapia gênica *ex vivo* 43;
3. Uso de genes de hematopoese: a hematopoese, a isquemia e a inflamação são processos biológicos intimamente interligados. A isquemia e a inflamação estimulam a hematopoese para produzir mais células sanguíneas, sendo os monócitos e os macrófagos os principais elementos que participam no controle de inflamação e na angiogênese^44^. As subpopulações dos monócitos e macrófagos podem ser classificadas, em função de suas atividades inflamatórias, em pró-inflamatórias e anti-inflamatórias^45^. E essas subpopulações podem promover ou inibir angiogênese e fibrogênese para reparo do tecido isquêmico e inflamado. Os genes que codificam fatores estimuladores de colônia como o fator estimulador de colônias de granulócitos e macrófagos (*granulocyte-macrophage colony-stimulating factor*, GM-CSF), o fator estimulador de colônias granulocitárias (*granulocyte colony-stimulating factor*, G-CSF) e o fator estimulante de colônias de macrófagos (*macrophage colony-stimulating factor*, M-CSF) e interleucinas como IL4 e IL13 participam no direcionamento e na ampliação dessas subpopulações de monócitos e macrófagos. Logo, o uso adequado desses genes poderá levar à formação e ao remodelamento de vasos de forma mais eficiente no ambiente isquêmico e inflamado^46^^,^^47^.

Em resumo, pode-se afirmar que a terapia gênica para isquemia de membro já é uma realidade clínica, visto que dois fármacos de terapia gênica já estão disponíveis no mercado. A eficácia dos fármacos vistos nesta revisão ainda é questionável, mas o importante é que o histórico de terapia gênica mostra que as pesquisas científicas e tecnológicas estão vencendo as barreiras de desconhecimentos e estão permitindo a criação de novos fármacos mais eficazes e seguros com materiais genéticos naturais ou sintéticos em associação com nanocarreadores ou vírus modificados geneticamente. No Brasil, a regulamentação de terapias avançadas, que inclui a terapia gênica, a terapia celular e a engenharia tecidual, foi aprovada pela Agência Nacional de Vigilância Sanitária (ANVISA) neste ano^48^. Com isso, os fármacos de terapia gênica já aprovados ou em fase de testes clínicos no exterior poderão ser comercializados ou testados no Brasil. Diante desse cenário, é importante que a medicina brasileira esteja preparada para usufruir dessa tecnologia e dos produtos por meio de atualizações.
